# Supplementary material for: Mutant CTNNB1 and histological heterogeneity define metabolic subtypes of hepatoblastoma
Source: EMBO Mol Med. 2017 Sep 19;9(11):1589–604. doi: 10.15252/emmm.201707814 (PMC5666308; doi:10.15252/emmm.201707814)
Supplement: Supplementary file 1 — Appendix [file EMMM-9-1589-s001.pdf]

Table of contents

Supplementary Methods

Supplementary References

Supplementary Figure Legends

Figure S1. Differential expression of glucose metabolism-associated genes between embryonal and fetal-like HB cells

Figure S2. YAP alterations impact on GLUT3

Figure S3. ChIP analysis for TCF4 or β-catenin enrichment at *GLUT3* gene

Figure S4. BCL9/BCL9L have minimal effect on GLUT3 expression

Figure S5. Sensitivity of human HB samples to 2-DG and 3BP

Supplementary Methods

RNA extraction, RT-PCR and real-time PCR

Total RNA was extracted using TRIzol Reagent (ThermoFisher) according to the manufacturer's instructions. DNAsi treatment was performed on RNA samples using Turbo DNA-free (Ambion). cDNA was synthesized from 1 μg total RNA using the high capacity reverse transcription kit (Applied Biosystems). Real-time PCR was performed with a StepOnePlus real-time PCR system (Applied Biosystems) using 5 ng of cDNA and commercially available Taqman probes for *SLC2A3* (Hs00359840), *CTNNB1* (s00355049_m1), *MYC* (Hs00153408_m1), *HK1* (Hs00175976_m1), *PFKP* (Hs00737354_m1), *LDHB* (Hs00929956_m1), *HK2* (Hs00606086_m1), *AQP9* (Hs00175573_m1), *GK* (Hs04235340_s1), *G6PC* (Hs02560787_s1), *PPARGC1A* (Hs01016719_m1), *BCL9* (Hs00979216_m1) and *BCL9L* (Hs00699441_m1). PCR data were normalized to levels of established housekeeping genes *GAPDH* (Hs02758991_g1), *ACTB* (Hs01060665_g1) and *TBP* (Hs00427620_m1). Raw data (Ct values) were analyzed according to the comparative Ct method. Briefly, three technical triplicates were used to validate and determine sample Ct mean. Ct means were processed as follows: ΔCt = (Ct gene of interest - Ct internal control); relative expression = 2−ΔCT sample A / 2−ΔCT sample B; whereby sample B serves for normalizing all samples to be compared.

Library preparation

Total RNA was obtained from 2 biological replicates for each cell line (HCC: Huh-1, Hep3B, HLE, HLF; HB: Hep-U2, Huh-6, HepG2, Hep293TT). RNA was isolated using

Trizol (Invitrogen). RNA quantities and quality were assessed using NanoDrop ND 1000 spectrophotometer and Advanced Analytical Fragment Analyzer.

RNA-seq libraries were prepared using the Illumina TruSeq Stranded mRNA reagents (Illumina; San Diego, California, USA) according to the protocol supplied by the manufacturer and using 200ng of total RNA. Cluster generation was performed with the resulting libraries using the Illumina TruSeq PE Cluster Kit v3 reagents and sequenced on the Illumina HiSeq 2000 using TruSeq SBS Kit v3 reagents. Sequencing data were processed using the Illumina Pipeline Software version 1.82.

Luciferase assay

HEK 293T cells were co-transfected with the specific luciferase reporter and constitutively active β-catenin expressing vector according to Lipofectamine 2000 manufacturer's protocol (Invitrogen). In luciferase reporter assays, a mix of 20 ng Renilla-Luciferase (phRL-TK, Promega, Madison, WI, USA), 200 ng of β-catenin plasmid and 200 ng luciferase reporter (promoter, intron 2, intron2_F1-F4, F4_a-c or intron 2-**Δ**- GTCTTTTGTTCTCA) was used for each well of a 12-well plate. Twenty-four hours after transfection, cells were lysed and relative light unit (RLU) was measured using the SpectraMax M5 luminometer (Molecular Devices) according to Dual-Luciferase Reporter Assay System manufacturer's protocol (Promega). The reported luciferase activity was the ratio between Firefly (GLUT3 construct)-Luciferase and Renilla-Luciferase, which was then normalized to 1 to get fold activities.

Immunocytochemistry and immunohistochemistry

Cells were fixed in 4% paraformaldehyde (PFA) for 10-30 min at RT and washed once with PBS. Cell pellet was resuspended in 500 μl 1% agar in PBS and allowed to harden in a mold for 10 min at RT. The obtained bloc was further fixed with 4% PFA O/N at 4°C, washed three times in PBS and dehydrated O/N. Human liver biopsies were fixed with formalin O/N at 4°C, washed 3x in PBS and dehydrated O/N. The day after, samples were embedded in paraffin, sectioned (4 μm) and mounted on slides. Following dewaxing and blocking of endogenous peroxidases with 1% hydrogen peroxide for 10 min at RT, antigen retrieval was done in a 10 mM Na-citrate buffer for 20 min at 95°C. Slides were washed 3x 5 min in PBS and blocked for 1 h at RT with 5% goat serum in PBS. Sections were stained with anti-GLUT3 (Calbiochem, #400062, 1:100), anti-Lin28 (Abcam, ab124765, 1:200), anti β-catenin (Abcam, ab32572, 1:250), anti-LDHB (Abcam, ab53292, 1:200) or anti-G6PC (Sigma, HPA052324, 1:100) antibodies O/N at 4°C. The day after, samples were washed in PBS and incubated with biotin-conjugated secondary antibodies for 1 h at RT. Avidin-biotin HRP complexes were added for 30 min (ABC kit, Vectastain), and the complexes were revealed with a DAB peroxidase substrate kit (Vector Laboratories, Burlingame, CA, USA). Counterstain was performed using Harris hematoxylin.

Human samples:

The HB specimens were provided by two hospitals. 13 cases from the Division of Clinical Pathology, Geneva University Hospitals, Switzerland and 20 from the Department of Pathology, Hôpital Bicêtre, HUPS, Assistance Publique-Hôpitaux de Paris, INSERM U1193, Faculté de Médecine Université Paris Sud, France.

Immunoprecipitation

Cells (8×10^7 per immunoprecipitation) were lysed in NP-40 buffer (0.2% NP-40, 150 mM NaCl, 20 mM Tris pH 8.0, 10 mM EDTA) containing a protease inhibitor cocktail (complete, Roche, Basel, Switzerland) and 1 mM Na_3VO_4 for 15 min on ice, followed by three quick steps of freezing in liquid N_2 and thawing at 37°C. Pre-clearing was achieved using sepharose-6B (Sigma-Aldrich, St. Louis, MO, USA) for 60 min at 4°C on a rotating wheel. Immunoprecipitation was performed using a 1:1 mixture of sepharose-6B and protein-G sepharose (Sigma-Aldrich), together with 2 μg control or specific antibody, overnight (O/N) at 4°C on a rotating wheel. After four steps of washing in lysis buffer, sample buffer was added, and the samples were boiled and loaded on a polyacrylamide gel for electrophoresis followed by Western blot. Antibodies used for immunoprecipitation were: anti-α-catenin (ab51032, Abcam) and anti-FLAG (F3135, Sigma-Aldrich).

Western Blotting

Except for nuclear-cytoplasmic fractionation, Western blots were performed on protein extract from cells lysed in RIPA buffer (20 mM Tris pH 8, 50 mM NaCl, 0.5% Na-deoxycholate, 0.1% SDS, 1 mM Na_3VO_4, protease inhibitor cocktail (complete, Roche)) at 4°C for 20 minutes. Following benzonase (Promega) treatment, samples were centrifuged for 15 min 14000 rpm at 4°C. Supernatant protein concentrations were determined by Pierce BCA Protein Assay Kit (Thermo Fisher Scientific). Lysates were resolved on SDS-PAGE under reducing conditions, and proteins were transferred to a PVDF membrane (Roche). For nuclear and cytoplasmic fractionation, cells were lysed

in EMSA Buffer A (20 mM Hepes pH 7.6, 20% glycerol, 10 mM NaCl, 1.5 mM $MgCl_2$, 0.2 mM EDTA, 1 mM DTT, 0.1% NP-40) 10 min at 4°C. The cytoplasmic fraction was collected after centrifugation for 5 min at 2500 rpm at 4°C. Following 3 times washing with EMSA Buffer A, nuclei were lysed in EMSA Buffer B (20 mM Hepes pH 7.6, 20% glycerol, 500 mM NaCl, 1.5 mM $MgCl_2$, 0.2mM EDTA, 1 mM DTT, 0.1% NP-40) 30 min at 4°C. Nuclear extracts were collected after centrifugation for 15 min at 13000 rpm at 4°C. The following antibodies were used: anti C terminal β-catenin (BD Transduction Laboratories, #610153, 1:1000), anti N-terminal β-catenin (Abcam, ab32572, 1:1000), anti-YAP (Cell Signaling, 4912, 1:1000), anti-β-tubulin (Santa Cruz Biotechnology, H-235, 1:2000), anti-PARP (Cell Signaling, 46D11, 1:1000), anti VSV (Sigma, P5D4, 1:1000) and anti FLAG (Sigma, M2, 1:1000). Western Lightning Plus ECL HRP substrate (Perkin Elmer) was used, according to manufacturer's instructions using X-ray medical films (Fujifilm).

Cell transfection

Transient inhibition of gene expression was performed by siRNA (Thermo Fisher) transfection. Cells plated onto a 6-well plate were transfected with 20 nM siRNA using RNAiMAX transfection reagent (Life Technologies) according to the manufacturer's instructions. To achieve a better MYC knockdown, a pool of four siRNAs was also used where indicated (Qiagen), kindly obtained from F. Radtke. Scrambled siRNA was used as control. Transient overexpression of cDNA was performed into HEK 293T (4 x 10^6) using Lipofectamine 2000 (15 μl). 10 μg of cDNA-expressing vector were used according to the manufacturer's instructions.

Mitotracker

Two different MitoTracker probes were used: the MitoTracker® Green probe allowing the detection of the entire mitochondrial mass, was used in the experiment to normalize for the mitochondrial content. In addition, a rosamine-based probe, the MitoTracker® Red, was used. This reduced probe does not fluoresce until it gets oxidized, reflecting the oxidation ability of the mitochondria. Cells were incubated at 37°C for 30 min with a pre-warmed staining solution containing 20 nM MitoTracker green FM (Invitrogen, M7515) and 100 nM MitoTracker Red CM-H_2-XRos (Invitrogen, M7514). Immediately after staining, cells were collected and analyzed by flow cytometry. Data were normalized to mitochondrial mass.

Annexin V staining

Cells were washed twice with PBS, and stained with Annexin V-APC antibody (Biolegend, #640012) diluted 1:200 into AnnexinV Binding Buffer (Biolegend, #422201) for 20 min at 4°C in the dark. Cells were centrifuged and resuspended into Annexin V Binding and DAPI (100 nM) prior to analysis. Apoptotic cells were determined using a Beckman Coulter CyAn ADP cytoflurometer. Both early (annexin V-positive, DAPI-negative) and late (annexin V-positive and DAPI-positive) apoptotic cells were included in cell death determinations.

Cloning

The promoter (chr12:8,086,713-8,089,712) and the intron 2 (chr12:8,068,995-8,103,194) of human *GLUT3* (*SLC2A3*) gene were used for luciferase assay (Masin et al, 2014). Intron 2 was further divided into 4 different fragments (F1-F4) and cloned upstream the luciferase reporter. The following primers were used to amplify F1 (For:

CTCGAGCTC ACTGGGGTCATCAATGCTCC, Rev: CTCCTCGAGGGGAGGGACCCTCACTAACT), F2 (For: CTCGAGCTCCAAACTAGGGGATTTTAAGA, Rev: CTCCTCGAGCCCAGCGTTCC GGGAGTAAG), F3 (For: CTCGAGCTCCGGTGCTGTTCTTCCTCCCA, Rev: CTCCTCG AGTCACAGTCTACCCCAGCCCT), F4 (For: CTCGAGCTCACATCGGTGCTGCCACC TAC, Rev: CTCCTCGAGGGTTGGTGGAAGAACAGAC) using intron 2 as PCR template.

After SacI and XhoI digestion, the F1-F4 were cloned into a luciferase reporter plasmid containing a minimal CMV promoter (kindly provided by J. Huelsken, EPFL, Lausanne) digested with the same restriction enzymes.

F4 was further fragmented into F4 a, b and c using the following primers: F4-a (For: CTCGAGCTCACATCGGTGCTGCCACC, Rev: CTCCTCGAGATAACGTATTGGAATTT ATG), F4-b (For: CTCGAGCTCGTTATCTGCCCCTTCCATTCC, Rev: CTCCTCGAGATT AGGGATCATTTCCTTCC), F4-c (For: CTCGAGCTCGGAAGGAAATGATCCCTAAT, Rev: CTCCTCGAGGGTTGGTGGAAGAACAGAC) and cloned into the luciferase reporter using the strategy described above.

Deletion of the TCF4 motif sequence (GTCTTTTGTTCTCA) was achieved by site-directed mutagenesis, using two different primer sets as follows: PCR1 (For: CTCGAGCTC ACTGGGGTCATCAATGCTCC, Rev: CTCCTCGAGGGTTGGTGGAAGAACAGACTGTTA CAGTTGGAAAACATTAGGGATCATGTT) and PCR2 (For: GGGTTAGTAAGCTGGGTT CCCTTAGC, Rev:

CTCCTCGAGGGTTGGTGGAAGAACAGAC). PCR products were combined in a second PCR interrupted after two amplification cycles and brought at 45°C for the addition of the original primer pair, following which PCR was resumed. Once sequenced to confirm the TCF4 deletion, intron 2 was cloned upstream the luciferase reporter using SacI and XhoI restriction sites.

The four β-catenin plasmids were cloned into a Met-VSV-MCS pCR3 expressing vector (kindly provided by P. Schneider, University of Lausanne). The mutated versions of β-catenin were obtained by site-directed mutagenesis of the wild-type β-catenin (*CTNNB1*) cDNA (kindly provided by J. Huelsken, EPFL, Lausanne), using two different primer sets as follows: Huh-6 β-catenin PCR1 (For: CTCGCGGCCGCTCATGGCTACTCAAGCTGATTTGA, Rev: AATGGATTAC AGAGTCCAGGTAAGAC), Huh-6 β-catenin PCR2 (For: GACTCTGTAATCCATTC TGGTGCCAC, Rev: CTCTCTAGATTACAGGTCAGTATCAAACCAG); HepG2 β-catenin PCR1 (For: CTCGCGGCCGCTCATGGCTACTCAAGCTGATTTG, Rev: AGTAGAACT ATCAAGTGACTAACAGCCGCTTT), HepG2 β-catenin PCR2 (For: CGGCTGTTAGT CACAACTATCAAGATGATGCA, Rev: CTCTCTAGATTACAGGTCAGTATCAAACCAG); Hep293TT β-catenin PCR1 (For: CTCGCGGCCGCTCATGGCTACTCAAGCTGATTTG, Rev: TTGATAGTTAATCACAGCCGCTTTTCTGTCTG), Hep293TT β-catenin PCR2 (For: CAGAAAAGCGGCTGTGATTAACTATCAAGATG, Rev: CTCTCTAGATTACAGGTCAG TATCAAACCAG). Hep-U2 β-catenin cDNA was obtained by single PCR using the following primers: For: CTC GCGGCCGCTCATGGCTACTCAAGATATTGATGGACAGTATG, Rev: CTCTCTAGATTACAGGTCAGTATCAAACCAG). PCR products were digested

using NotI and XbaI, cloned into the Met-VSV-MCS pCR3 vector digested with the same restriction enzymes and used for transfection.

Human *BCL9L* cDNA (purchased at the EPFL genomic platform) was amplified using the following primers: For: CTCGAATTCATGAGGATCCTGGCTAACAAGA, Rev: CTCCTCGAGGAAGGGCAGGTTGGCCATGCCG, digested with EcoRI and XhoI, and cloned into a Met-FLAG-MCS pCR3 expressing vector (P. Schneider, University of Lausanne). Human α-catenin (*CTNNA1*) cDNA (purchased at the EPFL genomic platform) was amplified using the following primers: For: CTCGAATTCATGACTGCTGTCCATGCAGGCA, Rev: CTCGCGGCCGCTTATTAGATGCTGTCCATAGCTTTG, digested with EcoRI and NotI, and cloned into Met-FLAG-MCS pCR3 expressing vector.

All PCRs for cloning were carried out using a high fidelity Taq polymerase contained in the Takara PrimeSTAR HS Premix (Clontech) according to manufacturer's protocol. YAP constructs were a kind gift from Joerg Huelsken and Oliver Hantschel (EPFL, Lausanne).

ATP production

Production of ATP was measured using the CellTiter-Glo®Luminescent Cell Viability Assay (Promega). Briefly, 10.000 cells/well were plated into a 96-well plate and treated with 40 μM 3BP. Cells cultured in normal medium were used as control. A volume of CellTiter-Glo® Reagent equal to the volume of medium present in each well was added and incubated 10 min on a shaker to induce cell lysis. The luminescent signal was measured after 15 min incubation at room temperature using the SpectraMax M5 luminometer (Molecular Devices). An ATP standard curve (1 μM, 100 nM, 10 nM, 1

nM) was generated on the same plate on which samples are assayed and used to calculate ATP concentration.

RNA sequencing statistic and bioinformatics

The RNA libraries were sequenced in two different runs, in order to assess the technical variability, which resulted largely smaller than the biological variability. Therefore, raw FASTQ sequences from technical replicates were merged in order to double the effective sequencing depth of each sample.

In order to address 3′ end adapter contamination, random RT primer artifacts, and 5′ end terminal-tagging oligonucleotide artifacts, raw FASTQ sequences were trimmed (Chen et al, 2014) with a base quality cut off of 5 and removing residual polyA tails and Illumina adapters. Trimmed FASTQ sequences were mapped with STAR (Dobin et al, 2013) to the human reference genome (Ensembl release, version GRCh37). Overall sequencing quality, absence of contaminants, and rRNA selection efficiency where assessed with the tools FASTqc (http://www.bioinformatics.babraham.ac.uk/projects/fastqc/) and NOIseqQC (Tarazona et al, 2011).

Gene expression has been assessed by RNA fragment counts obtained with featureCounts (Liao et al, 2014). We used the R analysis environment with the package edgeR (Robinson et al, 2010) to assess gene differential expression between the two groups of cell lines: embryonic and fetal. For a two groups design, we opted for testing for differential expression using edgeR's exact test, which is based on the quantile-adjusted conditional maximum likelihood method.

We have also investigated pathways enrichment, applying the ssGSEA algorithm (Barbie et al, 2009), which calculates separate enrichment scores for each pairing of a

sample and gene set. Each ssGSEA enrichment score represents the degree to which the genes in a particular gene set are coordinately up- or down-regulated within a sample. Gene sets were obtained from version 5 of the Molecular Signatures Database (MsigDB), collection C5 (Subramanian et al, 2005). As input to ssGSEA, RNA counts where normalized according to the library size and to the gene length providing RPKM normalized count matrix. The log2 RPKM matrix has been provided to the ssGSEA function of GenePattern (http://genepattern.broadinstitute.org/). ssGSEA enrichment scores were plotted on heatmaps and clustered with a k-means clustering algorithm.

Molecular modeling

The 3D structure of the human β-catenin, including residues encoded by exon 4, in complex with the human α-catenin was modeled from three experimental structures identified 2GL7 (Sampietro et al, 2006), 2Z6G (Xing et al, 2008) and 4ONS (Pokutta et al, 2014) in the protein databank (PDB) (Berman et al, 2000). 2GL7 contains the 3D structure of the human β-catenin/BCL9/Tcf4 complex. The first N-terminal residues of β-catenin resolved in this structure are "143-QDDAE[…]", immediately after exon 4. Exon 4 is thus absent from this structure. 4ONS provides the 3D structure of the complex between the mus musculus α- and β-catenins. The β-catenin residues resolved in this experimental structure expand from "83-DGQYA[…]" to "[…]LINYQ-143", and therefore covers most of exon 4, but not the following residues. The sequence identities are 72% and 100% between the human and mus musculus α-catenin and β-catenin residues resolved in the 4ONS structure, respectively. 2Z6G provides the crystal structure of the zebrafish β-catenin. The first N-terminal residues of β-catenin resolved in this structure are "127-EPSQMLKHAVVNLINYQDDAE[…]", thus including the C-

terminus of exon 4. Therefore, from a homology modeling point of view, the 2Z6G structure provides the link necessary to obtain a continuous 3D model of β-catenin joining exon 4 to the C-terminus of the protein. The sequence identity between the human and zebrafish β-catenin is 99%. Sequence alignments were performed using MUSCLE (Edgar, 2004). The model was obtained using the Modeller Program (Eswar et al, 2007; Sali & Blundell, 1993). 1000 models were generated by satisfaction of spatial restraints through minimization and simulated annealing, and the model with the best Modeller objective function was retained. Molecules were visualized and analyzed using UCSF Chimera (Pettersen et al, 2004).

NGS reads alignment and quality check

1. Sequencing quality check
    - Tool: FastQC
    - Version: 0.10.1
1. Cutting adapter sequences and low quality reads
    - Tool: cutadapt
    - Version: 1.4.2
    - Adapter sequences removes: PolyA tails (AAAAAAAAAAAA, TTTTTTTTTTTT), Illumina adapters (AGATCGGAAGAG, CTCTTCCGATCT)
    - Base quality cutoff 5
1. Alignment
    - Tool: STAR
    - Version: 2.4.0

- Reference genome: GRCh37

1. Sorting: reads are sorted by position in the reference genome
    - Tool: picard
    - Version: 1.96
1. Indexing: bam file is indexed for faster access
    - Tool: sambamba
    - Version: 0.4.7
1. Alignment statistics: number of aligned reads, multi-mapping reads, etc.
    - Tool: bamtools stats
    - Version: 2.3.0

Reads quantification and RNA-seq quality check

1. Reads counting: bam files are processed and the reads are assigned to transcripts
    - Tool: R package Rsubread (featureCounts function)
    - Version: 1.12.6
    - Multimapping reads : not counted
    - Multi-overlapping reads : not counted
    - Chimeric reads : not counted
    - Both ends mapped : not required
1. RNA-seq quality check. Different quantities are computed that allow to estimate the overall RNA-seq experiment quality
    - Tool: R package NOISeq
    - Version 2.6.0
    - coverage

- percentage of reads mapping to different genes biotypes
- fraction of rRNA

Differential gene expression

1. Differential gene expression. Differential expression is modeled by a general linear model (glm) algorithm, with the batch effect included in the model.
    - Tool: R package edgeR
    - Version: 3.4.2
    - Algorithm: glmFit

Design matrix: ~samples$batch+samples$condition

Supplementary references

Barbie DA, Tamayo P, Boehm JS, Kim SY, Moody SE, Dunn IF, Schinzel AC, Sandy P, Meylan E, Scholl C, Frohling S, Chan EM, Sos ML, Michel K, Mermel C, Silver SJ, Weir BA, Reiling JH, Sheng Q, Gupta PB et al (2009) Systematic RNA interference reveals that oncogenic KRAS-driven cancers require TBK1. Nature 462**:** 108-112

Berman HM, Westbrook J, Feng Z, Gilliland G, Bhat TN, Weissig H, Shindyalov IN, Bourne PE (2000) The Protein Data Bank. Nucleic Acids Res 28**:** 235-242

Chen C, Khaleel SS, Huang H, Wu CH (2014) Software for pre-processing Illumina next-generation sequencing short read sequences. Source Code Biol Med 9**:** 8

Dobin A, Davis CA, Schlesinger F, Drenkow J, Zaleski C, Jha S, Batut P, Chaisson M, Gingeras TR (2013) STAR: ultrafast universal RNA-seq aligner. Bioinformatics 29**:** 15-21

Edgar RC (2004) MUSCLE: multiple sequence alignment with high accuracy and high throughput. Nucleic Acids Res 32**:** 1792-1797

Eswar N, Webb B, Marti-Renom MA, Madhusudhan MS, Eramian D, Shen MY, Pieper U, Sali A (2007) Comparative protein structure modeling using MODELLER. Current protocols in protein science / editorial board, John E Coligan [et al] Chapter 2**:** Unit 2 9

Liao Y, Smyth GK, Shi W (2014) featureCounts: an efficient general purpose program for assigning sequence reads to genomic features. Bioinformatics 30**:** 923-930

Masin M, Vazquez J, Rossi S, Groeneveld S, Samson N, Schwalie PC, Deplancke B, Frawley LE, Gouttenoire J, Moradpour D, Oliver TG, Meylan E (2014) GLUT3 is induced during epithelial-mesenchymal transition and promotes tumor cell proliferation in non-small cell lung cancer. Cancer Metab 2**:** 11

Pettersen EF, Goddard TD, Huang CC, Couch GS, Greenblatt DM, Meng EC, Ferrin TE (2004) UCSF Chimera--a visualization system for exploratory research and analysis. J Comput Chem 25**:** 1605-1612

Pokutta S, Choi HJ, Ahlsen G, Hansen SD, Weis WI (2014) Structural and thermodynamic characterization of cadherin.beta-catenin.alpha-catenin complex formation. J Biol Chem 289**:** 13589-13601

Robinson MD, McCarthy DJ, Smyth GK (2010) edgeR: a Bioconductor package for differential expression analysis of digital gene expression data. Bioinformatics 26**:** 139-140

Sali A, Blundell TL (1993) Comparative protein modelling by satisfaction of spatial restraints. Journal of molecular biology 234**:** 779-815

Sampietro J, Dahlberg CL, Cho US, Hinds TR, Kimelman D, Xu W (2006) Crystal structure of a beta-catenin/BCL9/Tcf4 complex. Mol Cell 24**:** 293-300

Subramanian A, Tamayo P, Mootha VK, Mukherjee S, Ebert BL, Gillette MA, Paulovich A, Pomeroy SL, Golub TR, Lander ES, Mesirov JP (2005) Gene set enrichment analysis: a knowledge-based approach for interpreting genome-wide expression profiles. Proc Natl Acad Sci U S A 102**:** 15545-15550

Tarazona S, Garcia-Alcalde F, Dopazo J, Ferrer A, Conesa A (2011) Differential expression in RNA-seq: a matter of depth. Genome Res 21**:** 2213-2223

Xing Y, Takemaru K, Liu J, Berndt JD, Zheng JJ, Moon RT, Xu W (2008) Crystal structure of a full-length beta-catenin. Structure 16**:** 478-487

Supplementary Figure Legends

Appendix Figure S1. Differential expression of glucose metabolism-associated genes between embryonal and fetal-like HB cells. (A) Expression analysis (RPKM) of the indicated genes in the HB cell lines. (B) Real-time PCR analysis of *Hk1* in mouse embryonal (E13.5) and fetal livers (P0). Data show means ± s.d. (n=3) of *Hk1* expression relative to P0 liver. (C) Real-time PCR on MYC, GLUT3, PFKP, HK1 after

transfection with a pool of four siRNAs to knock down efficiently Myc, or siControl, in HepG2 cells (n=3).

Appendix Figure S2. YAP alterations impact on GLUT3. (A) Real-time PCR analysis for *GLUT3*, *PFKP*, *HK1* and *LDHB* expression in the indicated HB cell lines 72 h after transfection with control (white) or β-catenin (black) siRNA. Results are represented as relative expression compared to ctrl siRNA transfected cells. (B) Overexpression of YAP WT or YAP S127A (constitutively active form) was monitored by western blot (upper panel). GLUT3 expression was then analyzed by real-time PCR in the three HB cell lines (lower panel). (C) YAP silencing was monitored by western blot (upper panel). GLUT3 expression was then analyzed by real-time PCR in the three HB cell lines (lower panel). For A-C, data show means ± s.d. (n=3).

Appendix Figure S3. ChIP analysis for TCF4 or β-catenin enrichment at *GLUT3* gene. (A) and (B) Repetition n=2 and n=3 of the ChIP experiment described in Fig. 5F.

Appendix Figure S4. BCL9/BCL9L have minimal effect on GLUT3 expression. (A) Real-time PCR analysis for *BCL9* (black), *BCL9L* (grey) and *GLUT3* (white) expression in HB cell lines 72 h after transfection with control, or BCL9/BCL9L siRNA. Results are represented as relative expression compared to control (ctrl) siRNA-transfected cells. The expression of *GLUT3* is faintly reduced upon *BCL9*/*BCL9L* inhibition in Huh-6. (B) Luciferase activity was measured in HEK 293T cells 24 h after transfection with the intron 2 (I2) of *GLUT3* reporter vector, *CTNNB1* alone or together with *BCL9L*. Cells transfected with an empty luciferase reporter were used as control. Results are

represented as relative light unit (RLU) compared to control. For A-B, data show means ± s.d. (n=3).

Appendix Figure S5. Sensitivity of human HB samples to 2-DG and 3BP. (A) Repetition of caspase-3/7 assay described in Fig. 6B. (B) Representative flow cytometry plot for the indicated cell lines stained with Annexin-V after treatment with 2-DG. (C) ATP production quantification after 2-DG treatment in hepatoblastoma cell lines. (D) Representative flow cytometry plot for the indicated cell lines stained with Annexin-V after treatment with 3BP. (E) Caspase 3/7 activity after 3BP treatment in hepatoblastoma cell lines.

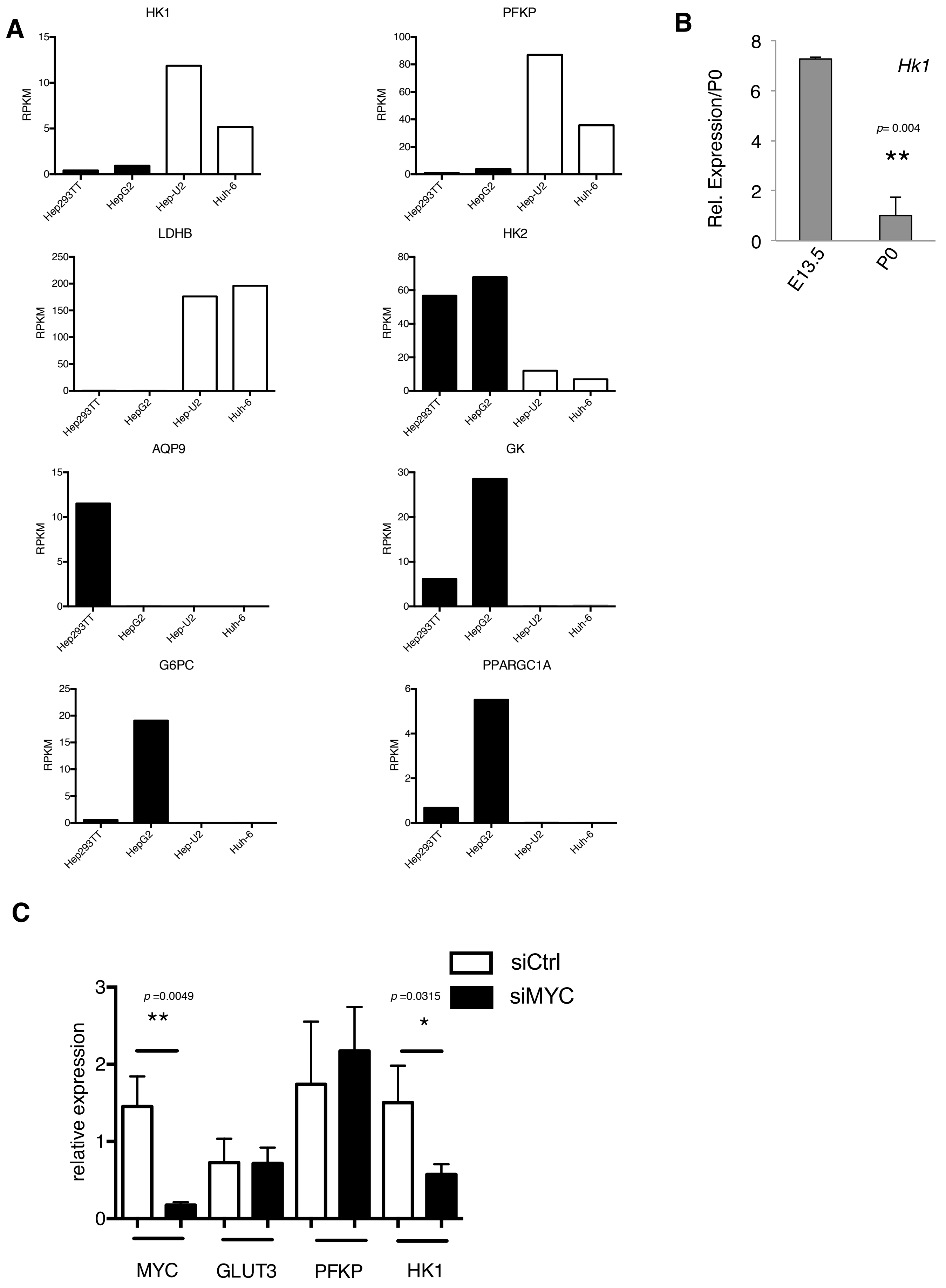

Appendix Figure S1

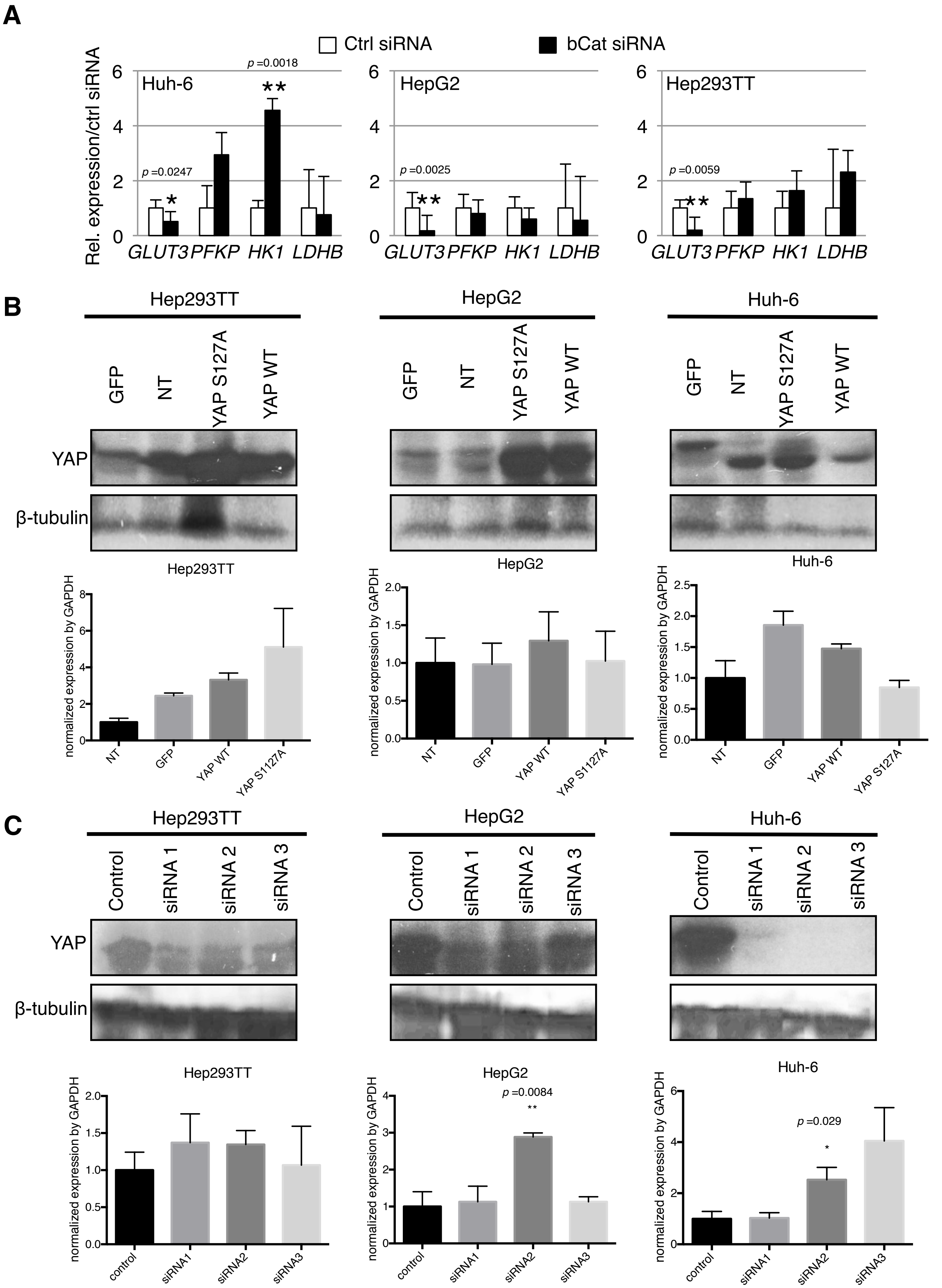

Appendix Figure S2

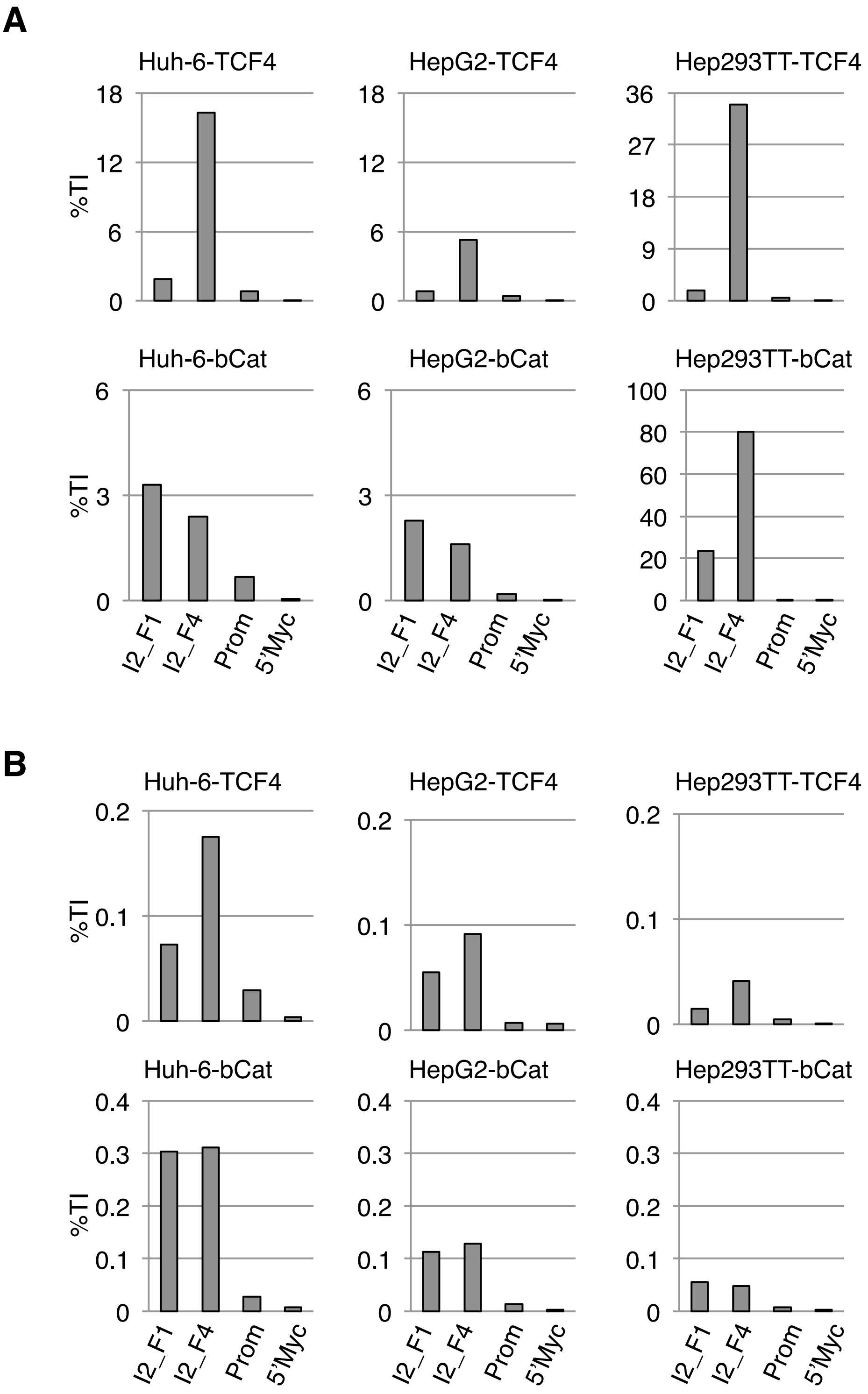

Appendix Figure S3

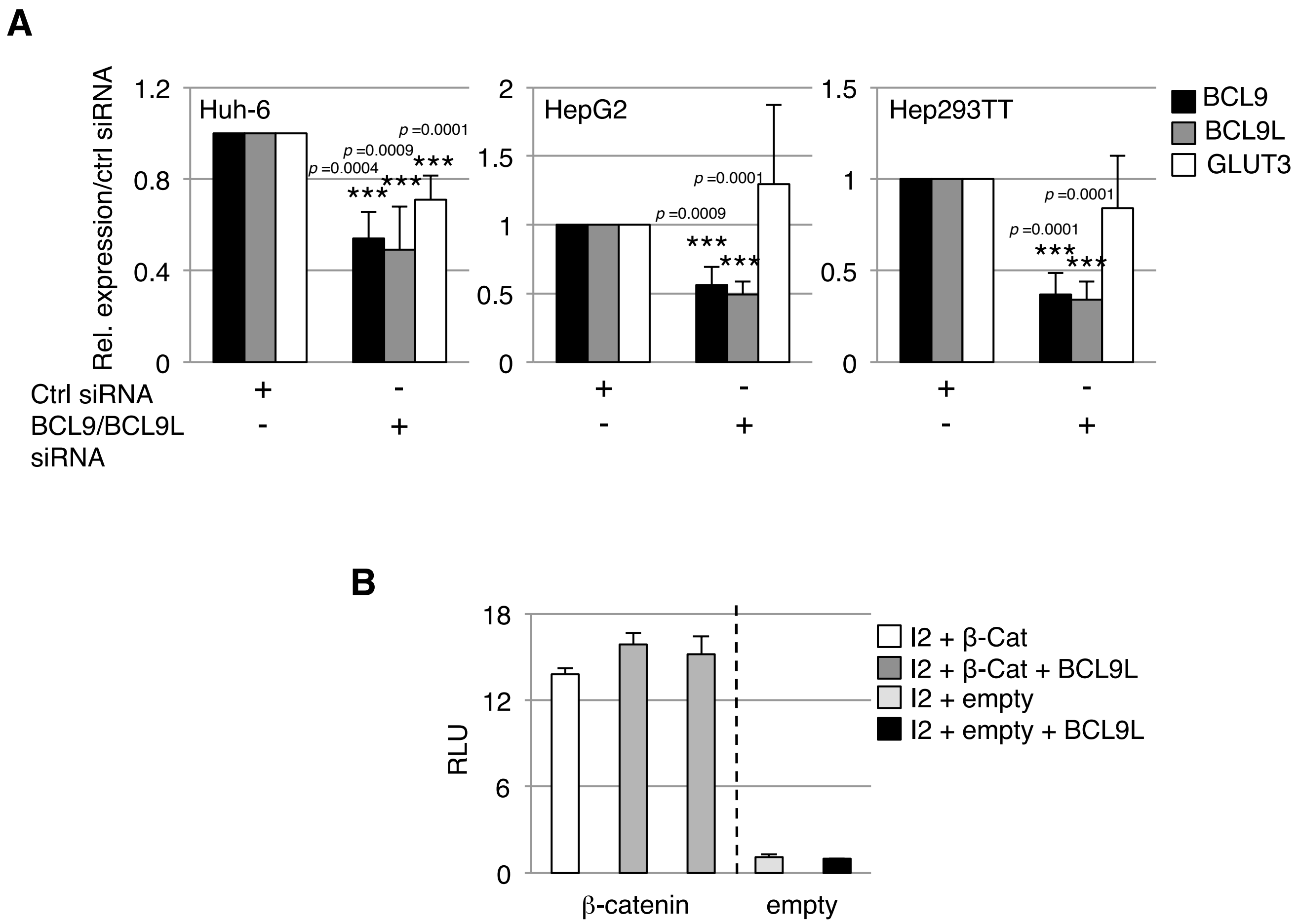

Appendix Figure S4

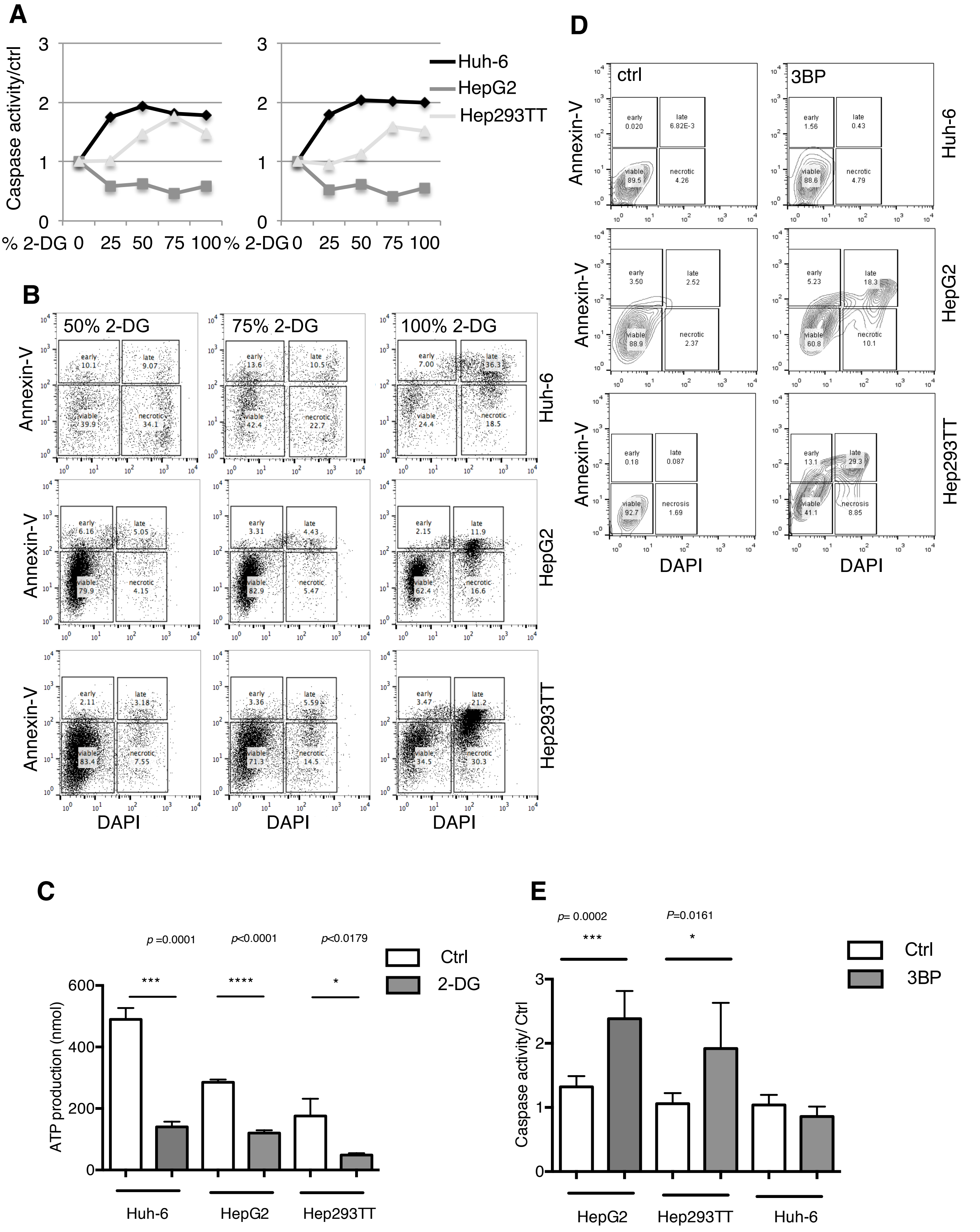

Appendix Figure S5
