# Supplementary material for: Mutant CTNNB1 and histological heterogeneity define metabolic subtypes of hepatoblastoma
Source: EMBO Mol Med. 2017 Sep 19;9(11):1589–604. doi: 10.15252/emmm.201707814 (PMC5666308; doi:10.15252/emmm.201707814)
Supplement: Supplementary file 2 — Expanded View Figures PDF [file EMMM-9-1589-s002.pdf]

Expanded View Figures

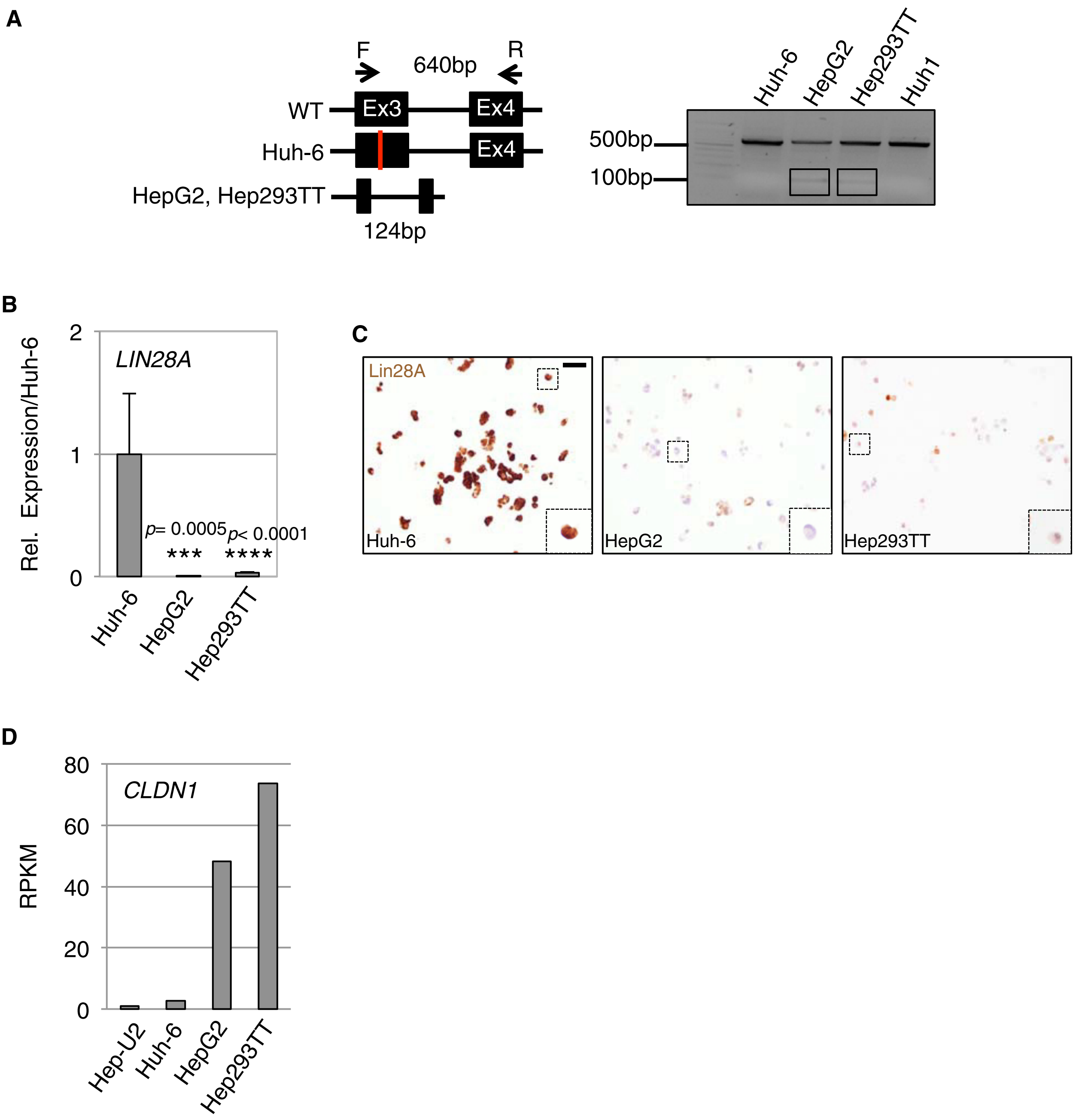

Figure EV1. Molecular characterization of embryonal and fetal-like HB cells.

A Schematic representation of the primers used to amplify the genomic region spanning exon 3 to exon 4 of the *CTNNB1* gene (left panel). Genomic DNA was isolated from the indicated cells lines and amplified by PCR. A PCR product of 640 bp was obtained from the full-length *CTNNB1* gene. The appearance of a 124-bp band in HepG2 and Hep293TT indicated a heterozygous deletion of *CTNNB1* gene (right panel).

B Real-time PCR analysis for the expression of *LIN28A* in the indicated cell lines. Data show means ± s.d. (n = 3). P-values were determined by Mann–Whitney test.

C Immunocytochemistry of Lin28A on the indicated cell lines. Dashed squares highlight a zoom on a cell. Scale bar: 20 μm.

D Expression analysis (RPKM) of the fetal marker claudin-1 (*CLDN1*) in the HB cell lines.

© 2017 The Authors

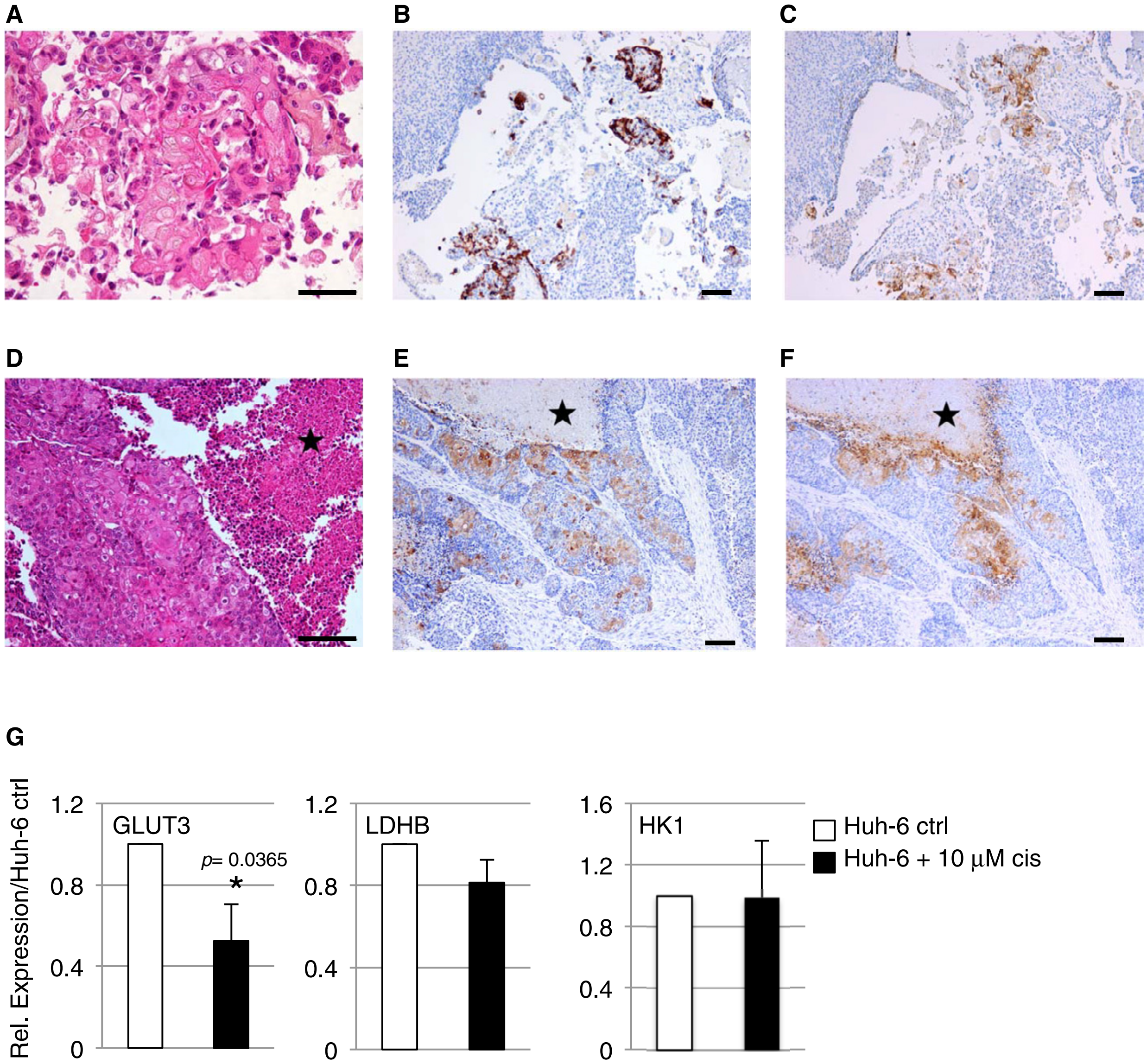

Figure EV2. Representative histological and immunohistochemistry findings.

A–F GLUT3 reactivity was observed in the embryonal and squamous components only. Standard HPS stain shows squamous differentiation foci (A), confirmed by a CK5/6 immunostain (B and E). In the two tumors shown (C and F), focal mild 1+ cytoplasmic, and moderate 2+ membranous GLUT3 reactivity is seen. The embryonal component of the second tumor (D-F) shows a serpiginous architecture, with foci of tumor necrosis (star). Scale bars = 100 µm, except for (A) (scale bar = 50 µm).

G Relative expression of *GLUT3*, *LDHB*, and *HK1* in Huh-6 cells treated or not for 24 h with cisplatin (cis). Data show means ± s.d. (n = 4). P-values were determined by Mann–Whitney test.

Figure EV3. α-Catenin interacts with short but not long deletion HB mutant β-catenin.

A Ribbon representation of the modeled 3D structure of human β-catenin in complex with human α-catenin. Residues that are absent in HepG2 and Hep293TT are in dark blue, and are responsible for binding to α-catenin. The experimental binding position of BCL9 on β-catenin is shown in pink for information. However, this model does not presume that BCL9 and α-catenin are able to bind concomitantly to β-catenin.

B Co-immunoprecipitation using protein extracts from 293T cells transfected with the indicated mutant forms of VSV-β-catenin and FLAG-α-catenin constructs or empty-FLAG as negative control. Proteins were immunoprecipitated using anti-FLAG antibody.

C Protein extracts from the indicated cell lines were used for endogenous α-catenin or control IgG immunoprecipitation, followed by Western blot using the indicated antibodies. The arrowheads indicate the position of the full-length or deleted (Δex3-4) forms of β-catenin. TI, total input; IP, immunoprecipitation.

Source data are available online for this figure.

▶

 © 2017 The Authors

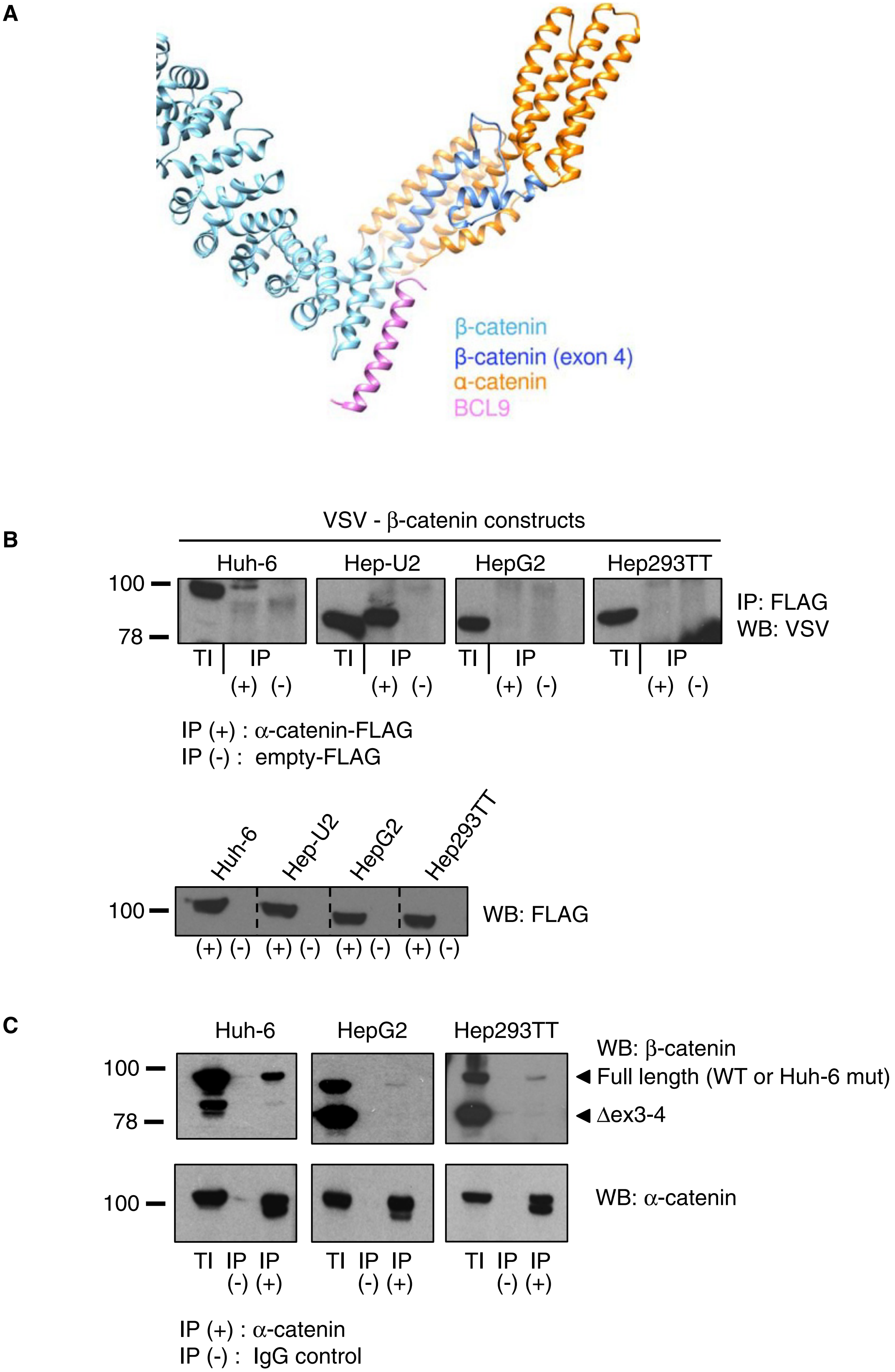
A
β-catenin
β-catenin (exon 4)
α-catenin
BCL9
B
VSV - β-catenin constructs
Huh-6
Hep-U2
HepG2
Hep293TT
100
78
IP: FLAG
WB: VSV
TI IP
(+) (-)
IP (+) : α-catenin-FLAG
IP (-) : empty-FLAG
Huh-6
Hep-U2
HepG2
Hep293TT
100
WB: FLAG
(+) (-) (+) (-) (+) (-) (+) (-)
C
Huh-6
HepG2
Hep293TT
WB: β-catenin
Full length (WT or Huh-6 mut)
Δex3-4
WB: α-catenin
TI IP IP
(-) (+)
IP (+) : α-catenin
IP (-) : IgG control

Figure EV3.
