# Supplementary material for: Mutant CTNNB1 and histological heterogeneity define metabolic subtypes of hepatoblastoma
Source: EMBO Mol Med. 2017 Sep 19;9(11):1589–604. doi: 10.15252/emmm.201707814 (PMC5666308; doi:10.15252/emmm.201707814)

Figure EV3B: IP FLAG, VSV staining.

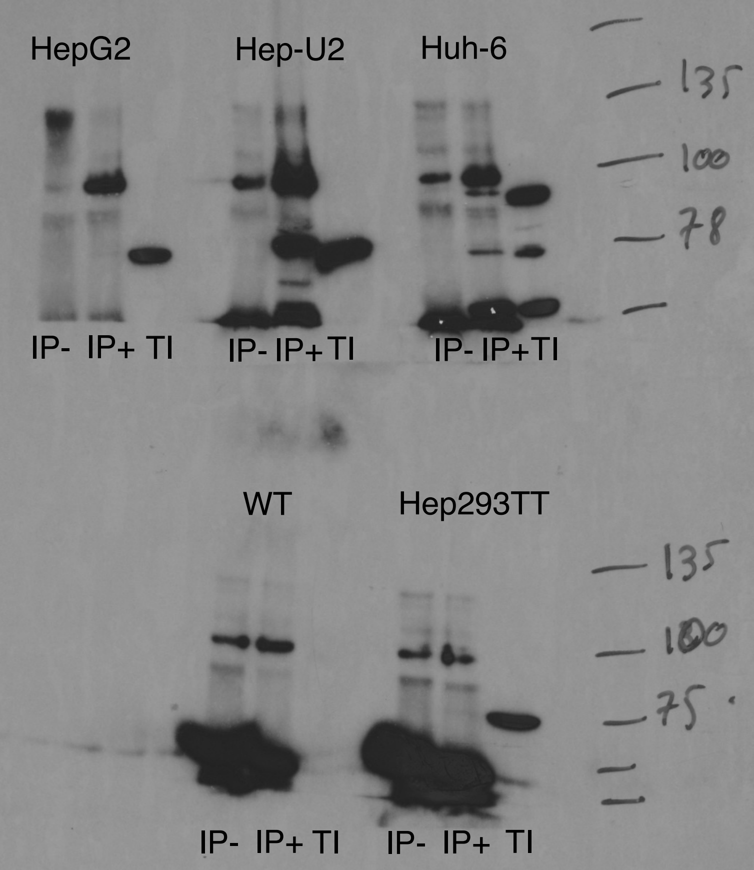

Figure EV3B: IP FLAG, FLAG staining.

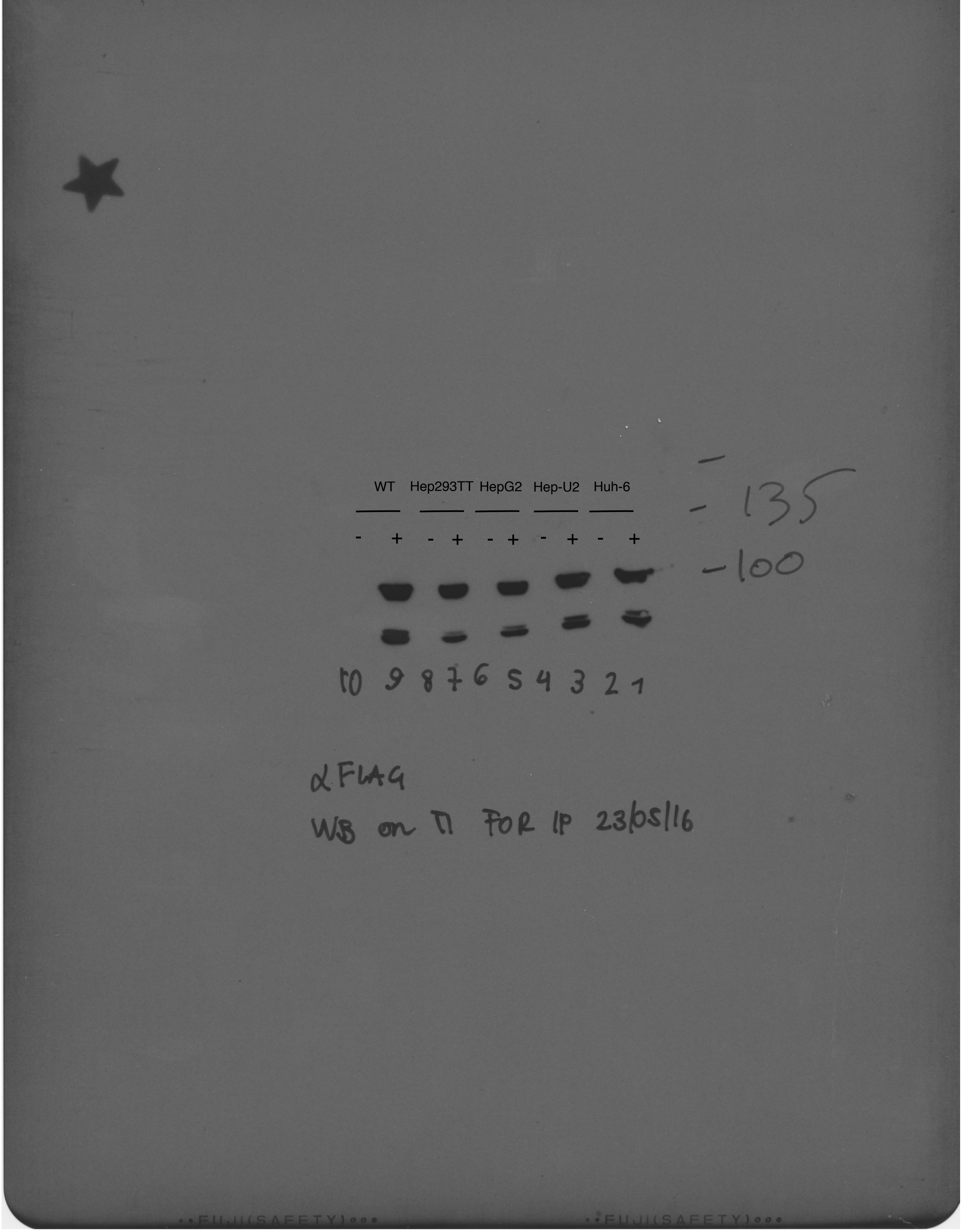

Figure EV3C: IP α-catenin, WB α-catenin

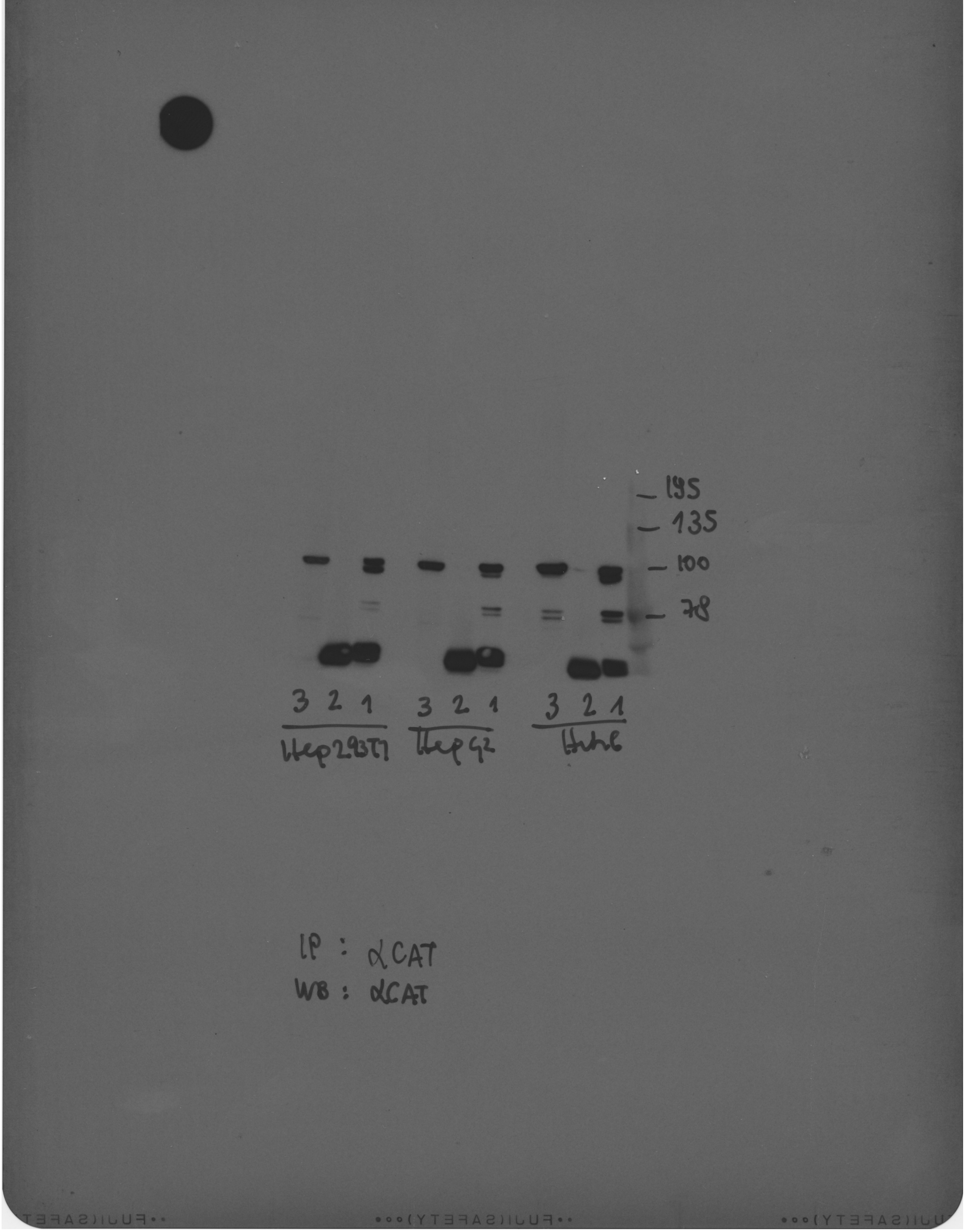

Figure EV3C: IP α-catenin, WB β-catenin

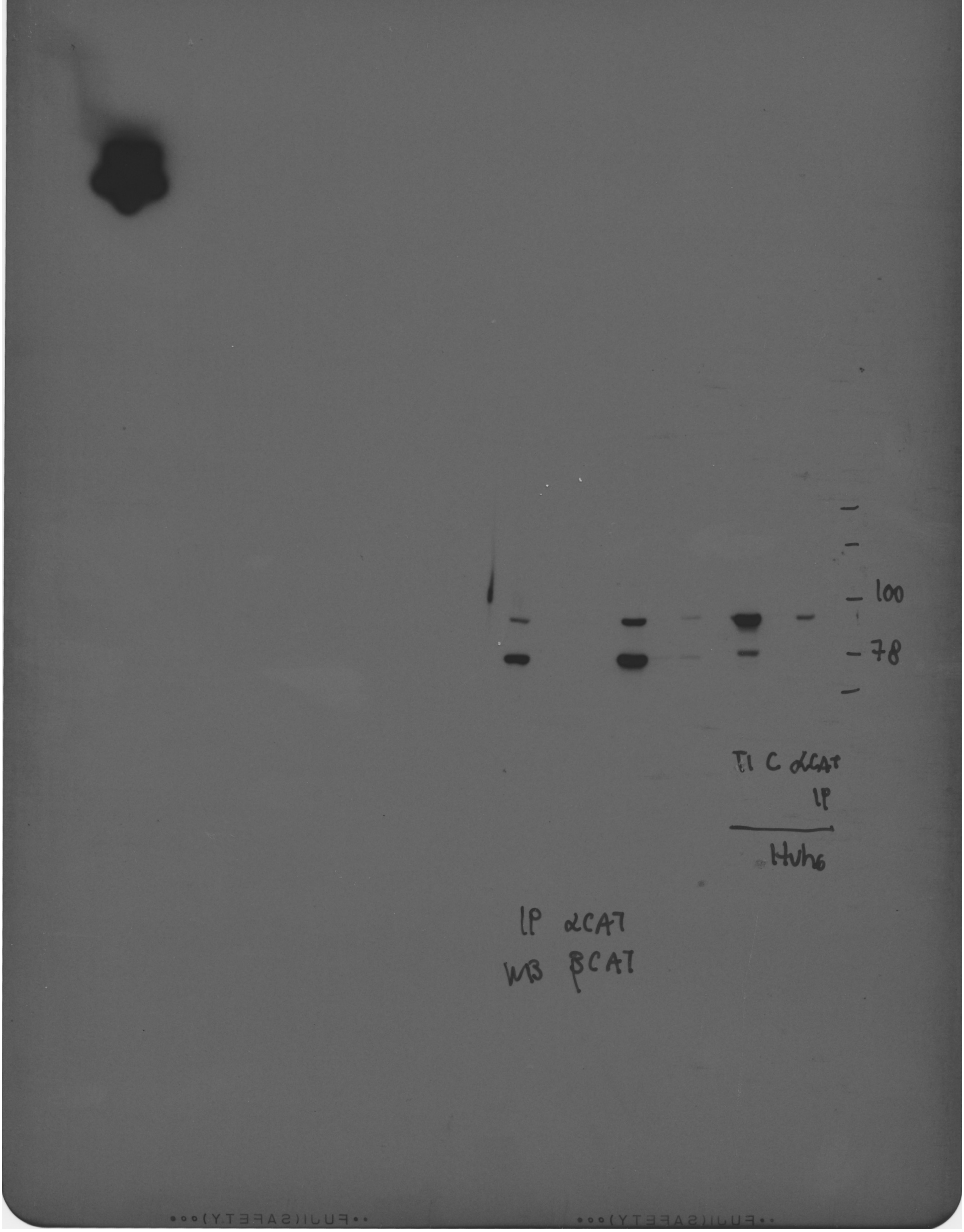

Figure EV3C: IP α-catenin, WB β-catenin

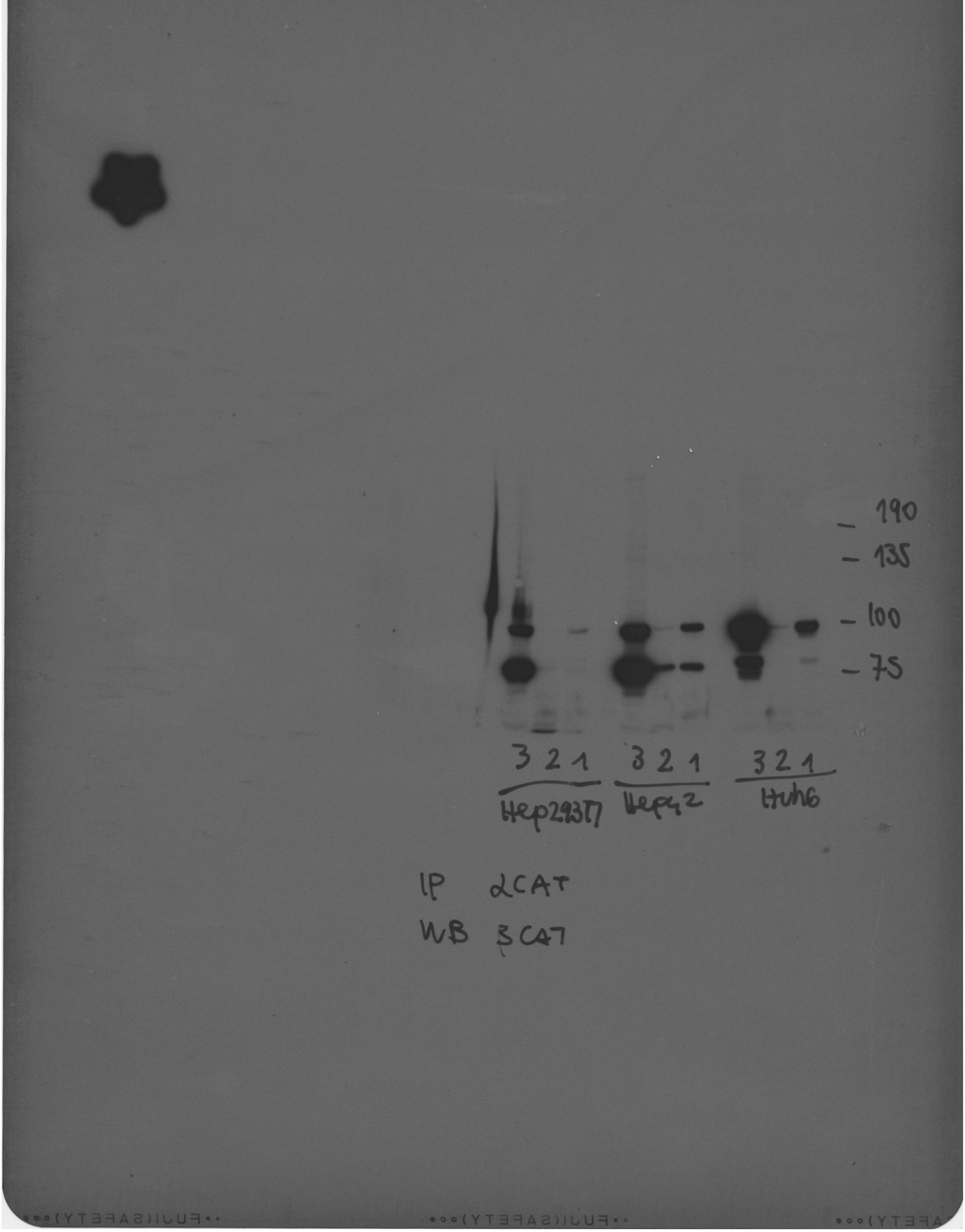

Supplement: Supplementary file 8 — Source Data for Figure EV3 [file EMMM-9-1589-s009.pdf]
