# Supplementary material for: Mutant CTNNB1 and histological heterogeneity define metabolic subtypes of hepatoblastoma
Source: EMBO Mol Med. 2017 Sep 19;9(11):1589–604. doi: 10.15252/emmm.201707814 (PMC5666308; doi:10.15252/emmm.201707814)

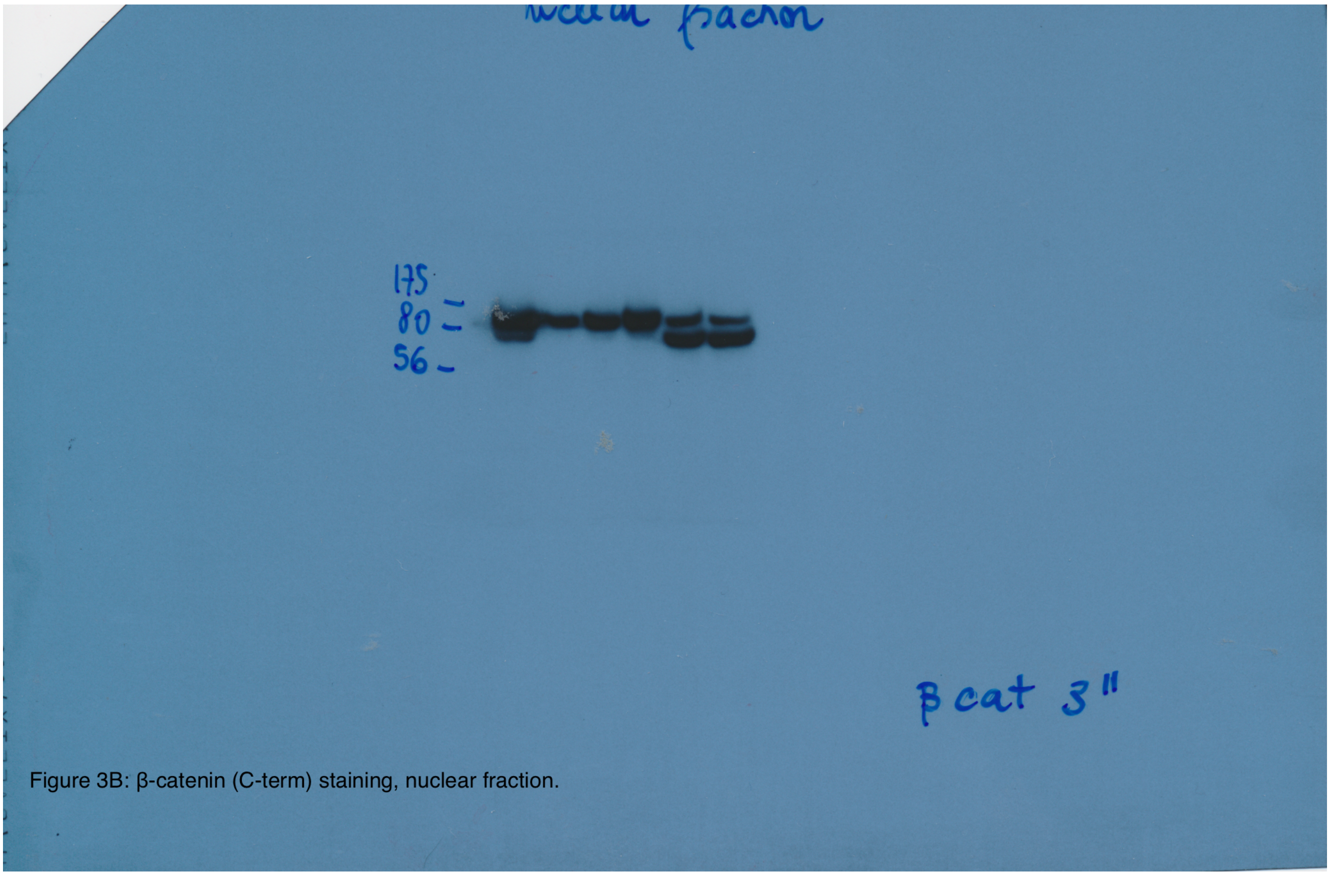

Figure 3B: β-catenin (C-term) staining, nuclear fraction.

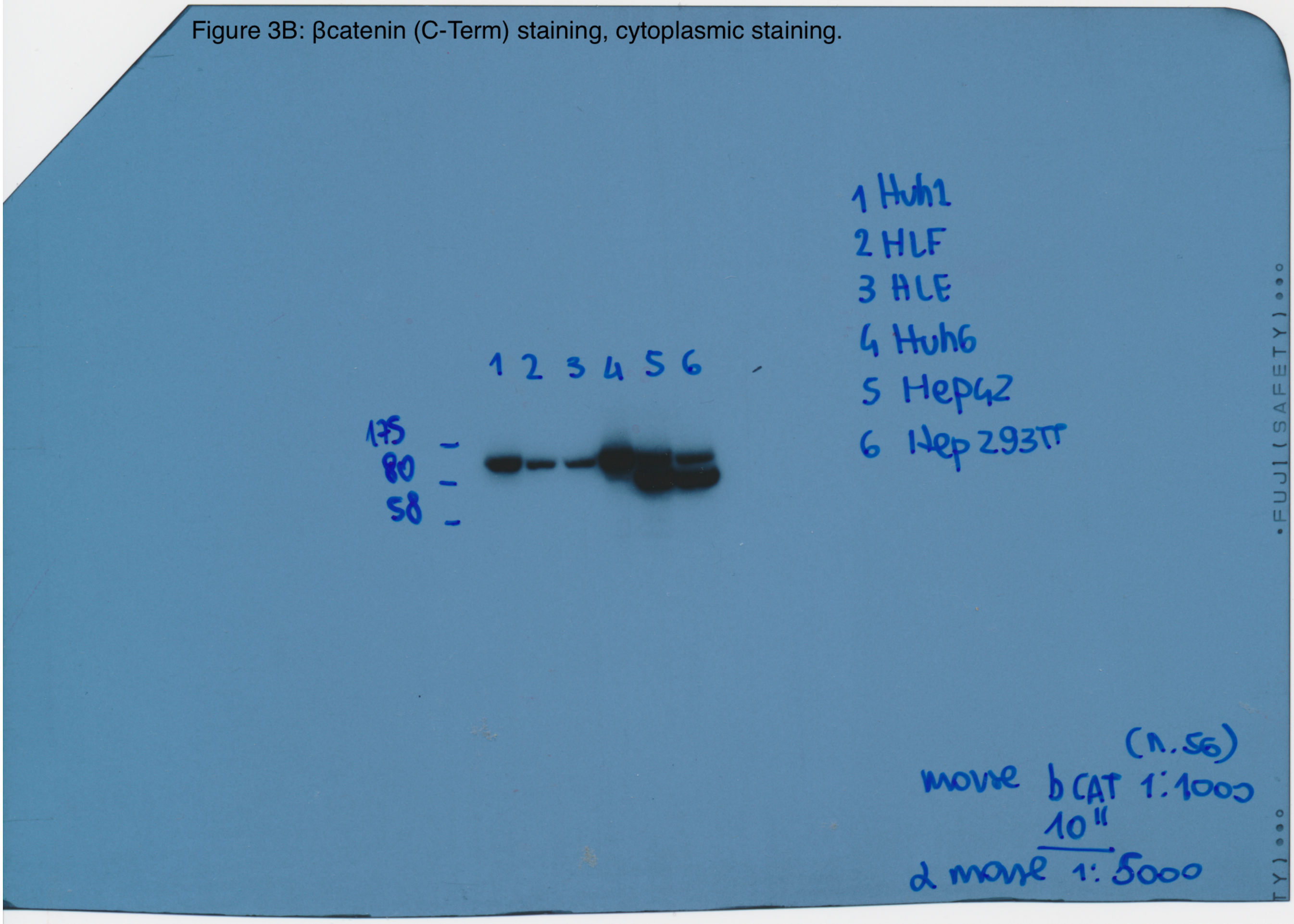

Figure 3B: βcatenin (C-Term) staining, cytoplasmic staining.

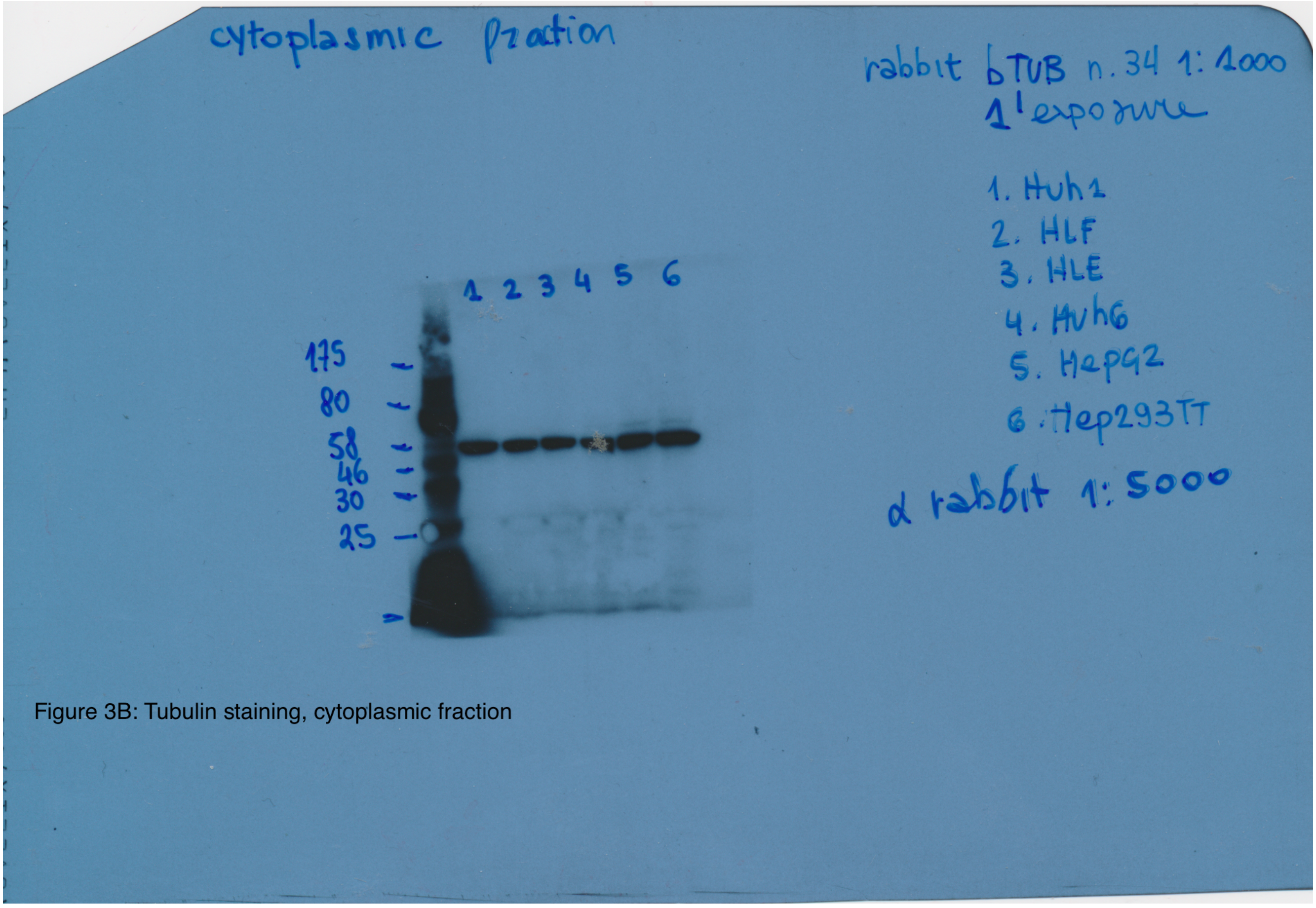

Figure 3B: Tubulin staining, cytoplasmic fraction

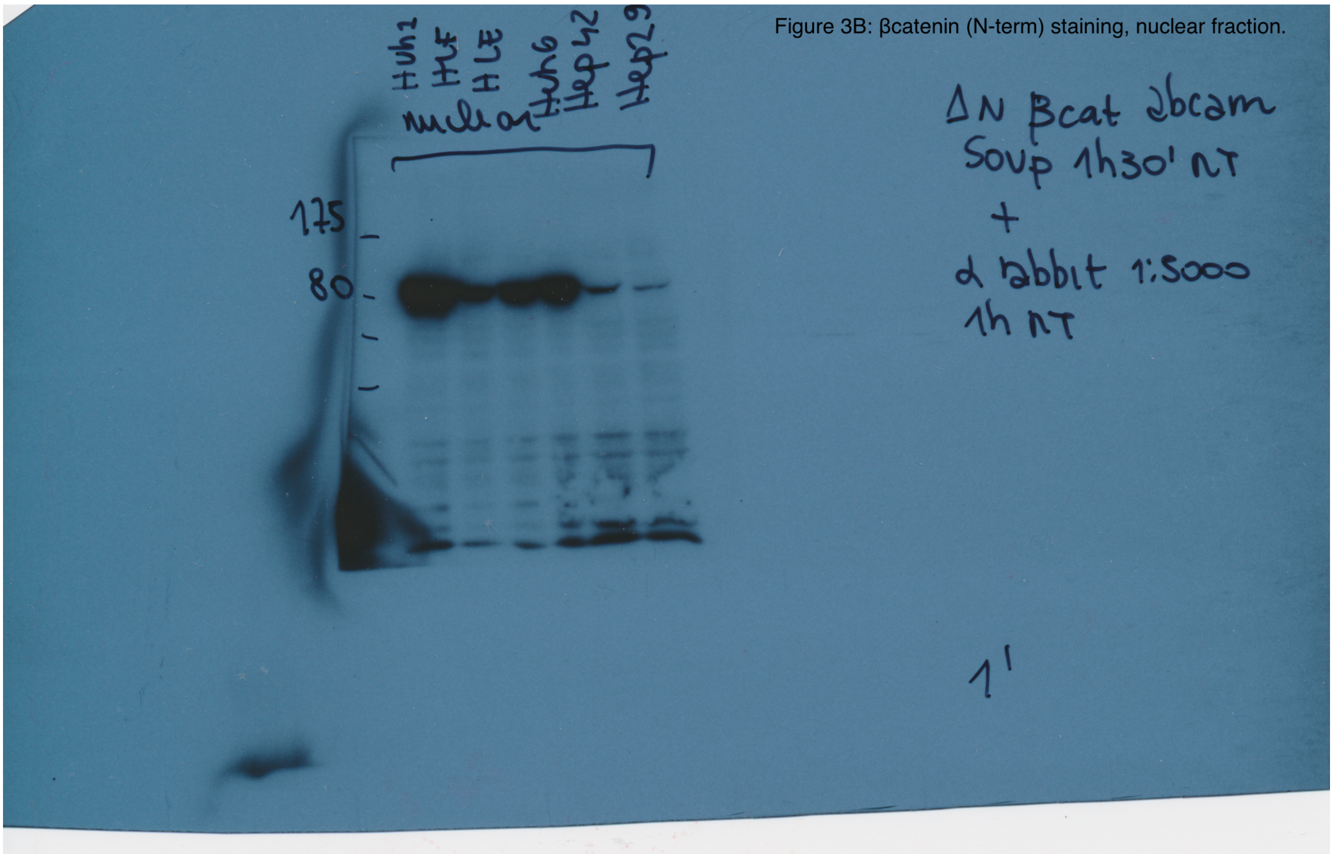

Figure 3B: βcatenin (N-term) staining, nuclear fraction.

Figure 3B: βcatenin (N-term) staining, cytoplasmic fraction.

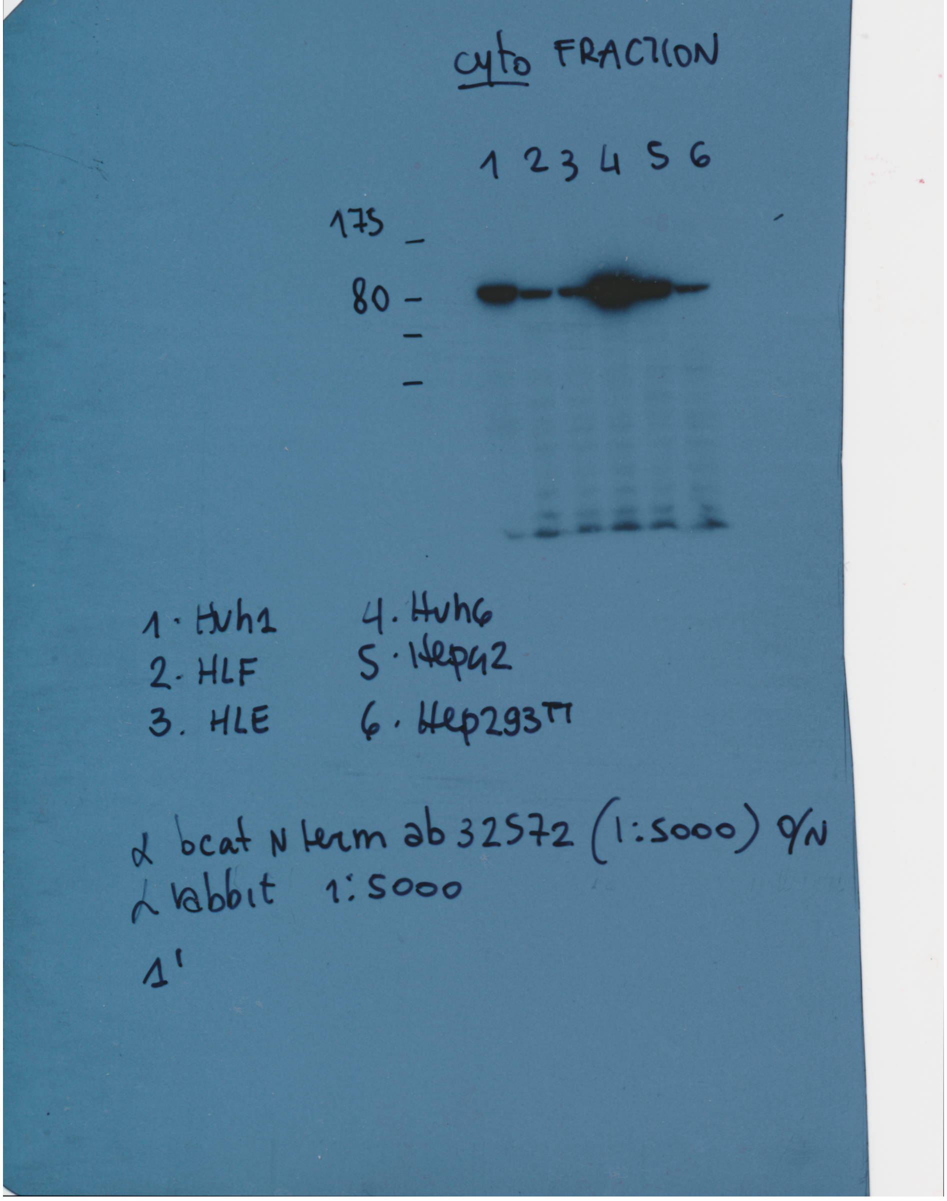

Figure 3B: PARP staining, nuclear fraction.

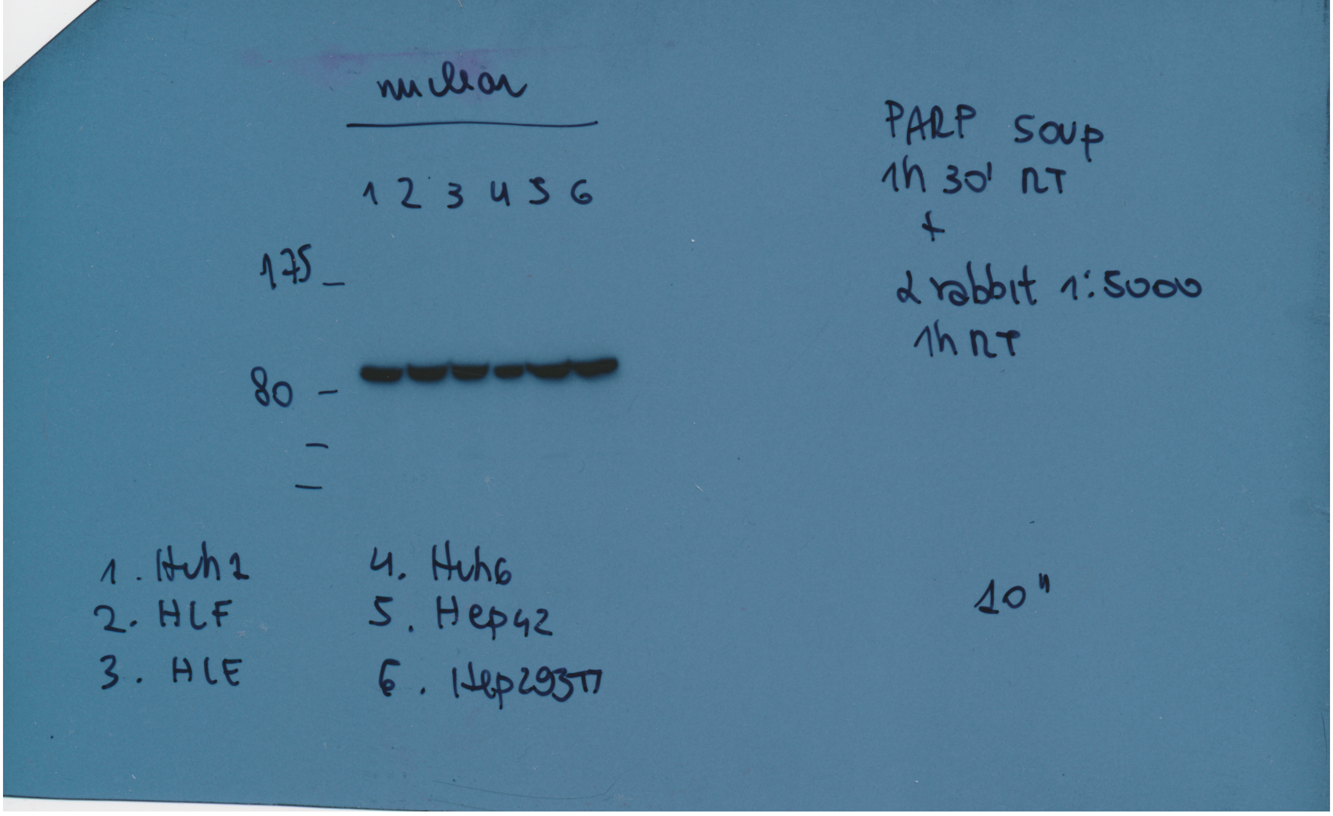

Supplement: Supplementary file 10 — Source Data for Figure 3 [file EMMM-9-1589-s008.pdf]
